# Supplementary material for: An endothelial activin A-bone morphogenetic protein receptor type 2 link is overdriven in pulmonary hypertension
Source: Nat Commun. 2021 Mar 19;12:1720. doi: 10.1038/s41467-021-21961-3 (PMC7979873; doi:10.1038/s41467-021-21961-3)
Supplement: Supplementary file 1 — Supplementary Information [file 41467_2021_21961_MOESM1_ESM.pdf]

Supplementary Information

An endothelial activin A-bone morphogenetic protein receptor type 2 link is overdriven in pulmonary hypertension

Ryanto et al.

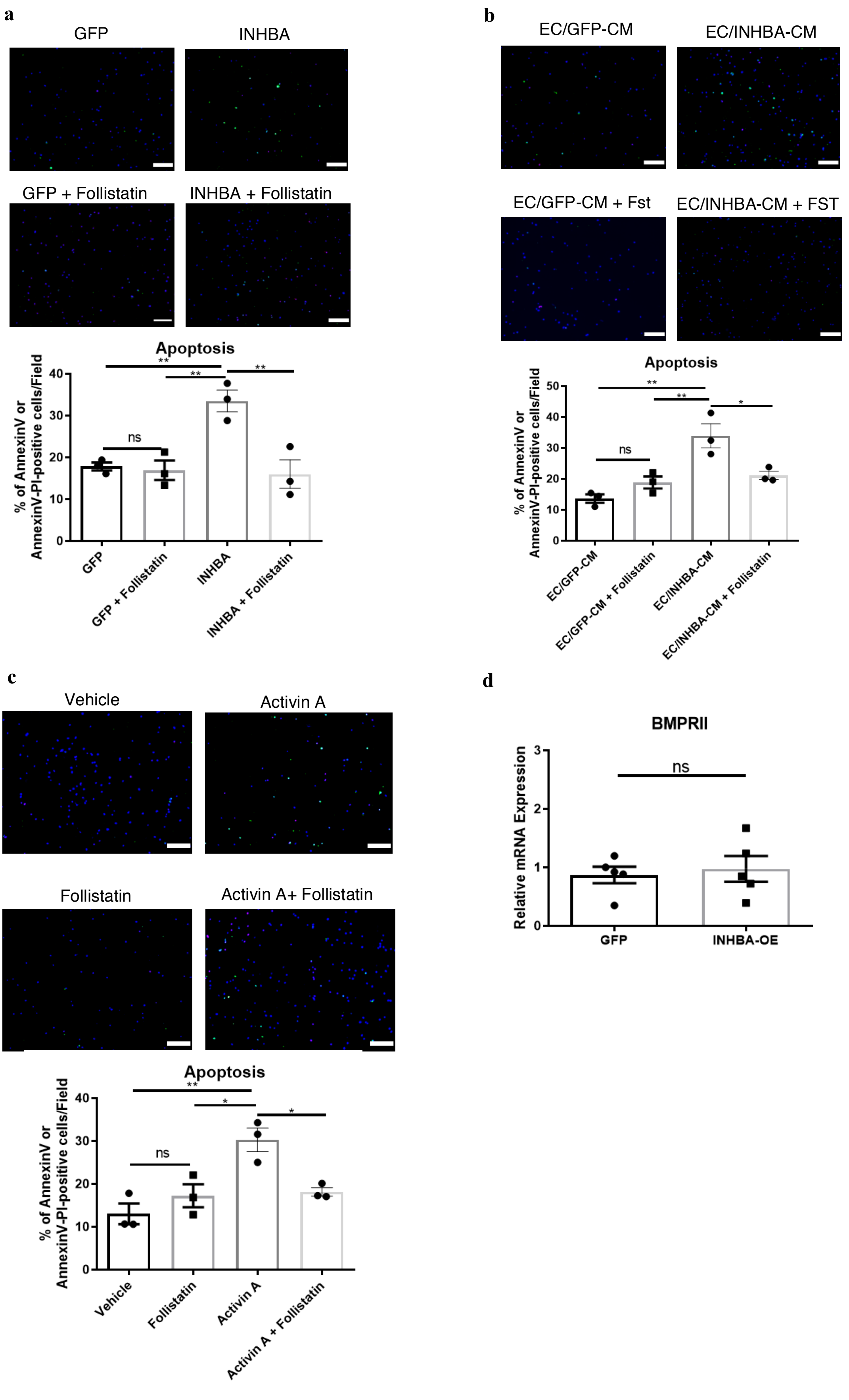

a
GFP
INHBA
GFP + Follistatin
INHBA + Follistatin
Apoptosis
% of AnnexinV or AnnexinV-PI-positive cells/Field
ns
**
GFP
GFP + Follistatin
INHBA
INHBA + Follistatin
b
EC/GFP-CM
EC/INHBA-CM
EC/GFP-CM + Fst
EC/INHBA-CM + FST
Apoptosis
% of AnnexinV or AnnexinV-PI-positive cells/Field
ns
**
*
EC/GFP-CM
EC/GFP-CM + Follistatin
EC/INHBA-CM
EC/INHBA-CM + Follistatin
c
Vehicle
Activin A
Follistatin
Activin A+ Follistatin
Apoptosis
% of AnnexinV or AnnexinV-PI-positive cells/Field
ns
**
*
Vehicle
Follistatin
Activin A
Activin A + Follistatin
d
BMPRII
Relative mRNA Expression
ns
GFP
INHBA-OE

Supplementary Fig. 1. INHBA/ActA exacerbates endothelial apoptosis.
a, Representative images and quantitation of EC apoptosis induced by serum and growth factor depletion, assessed by Annexin-V (green) /PI (red) staining (n = 3 in each group). PAECs transfected with either GFP or INHBA were examined in the presence or absence of 100 ng/mL recombinant Follistatin (FST). **b**, Representative images and quantitation of EC apoptosis induced by 200 nM H_2O_2, assessed by Annexin-V (green) /PI (red) staining. PAECs treated with conditioned medium (CM) derived from PAECs transfected with either GFP or INHBA were examined in the presence or absence of 100 ng/mL recombinant FST (n = 3 in each group). **c**, Representative images and quantitation of EC apoptosis (n = 3 in each group) induced by serum and growth factor depletion, assessed by Annexin-V (green) /PI (red) staining. PAECs treated with vehicle or ActA (20 ng/mL) were examined in the presence or absence of 100 ng/mL recombinant Follistatin. Bars: 100 μm. **d,** Quantitative PCR analysis for BMPRII in PAECs transfected with either GFP or INHBA (INHBA-OE) (n = 3 in each group). **P* < 0.05, ***P* < 0.01, ****P* < 0.001, *****P* < 0.0001, n.s., not significant (P > 0.05). Exact P values are shown in the Source Data file. Data are presented as the mean ± SEM. One-way ANOVA with Tukey's post-hoc test was used to compare between groups (a–c), while two-sided student's *t*-test was used to compare between the groups (d).

a

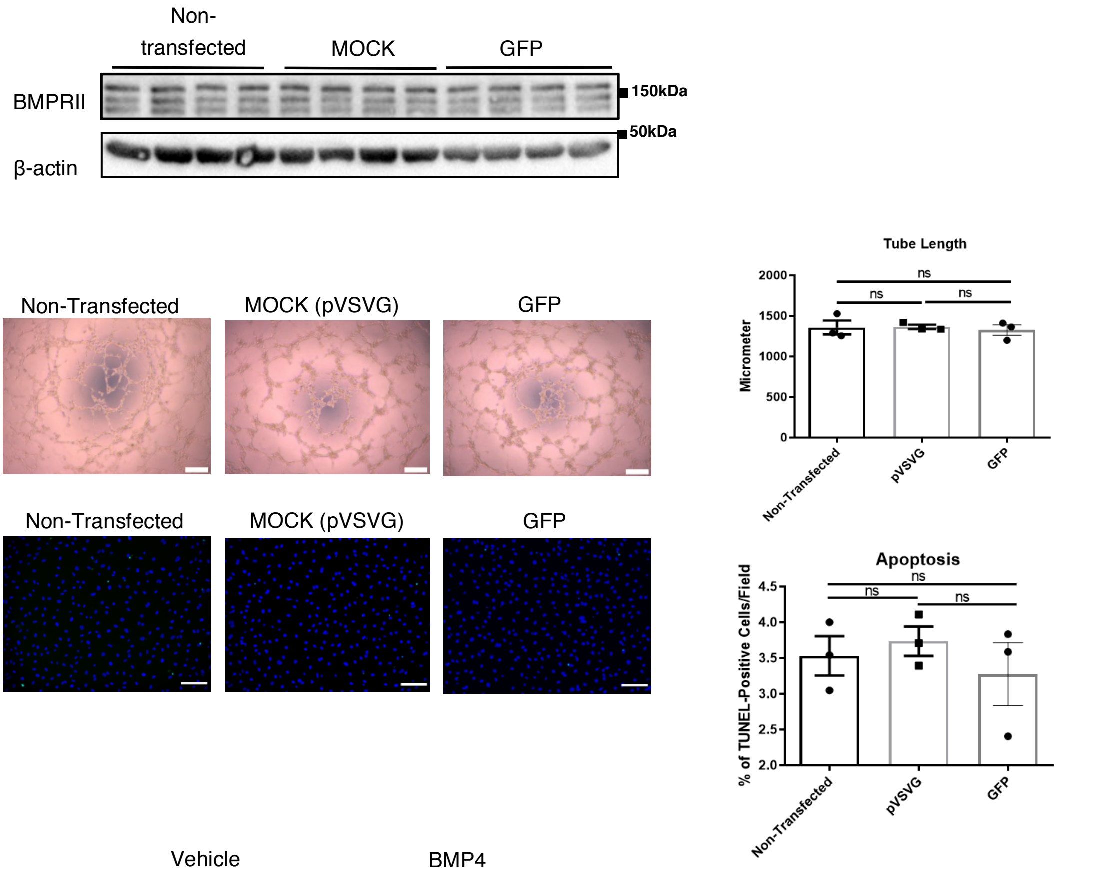
Non-
transfected
MOCK
GFP
BMPRII
150kDa
50kDa
β-actin
Non-Transfected
MOCK (pVSVG)
GFP
Non-Transfected
MOCK (pVSVG)
GFP
Tube Length
Micrometer
2000
1500
1000
500
0
ns
ns
ns
Non-Transfected
pVSVG
GFP
Apoptosis
% of TUNEL-Positive Cells/Field
4.5
4.0
3.5
3.0
2.5
2.0
ns
ns
ns
Non-Transfected
pVSVG
GFP

b

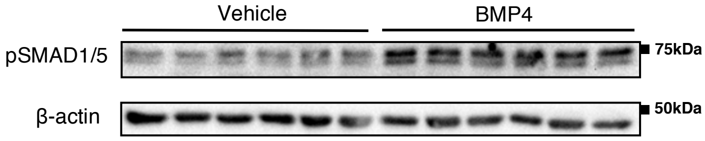
Vehicle
BMP4
pSMAD1/5
75kDa
β-actin
50kDa

c

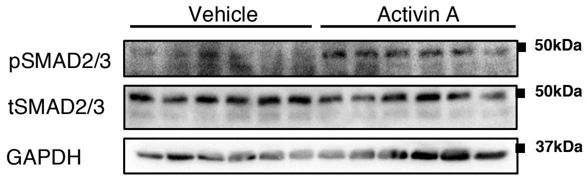
Vehicle
Activin A
pSMAD2/3
50kDa
tSMAD2/3
50kDa
GAPDH
37kDa

d

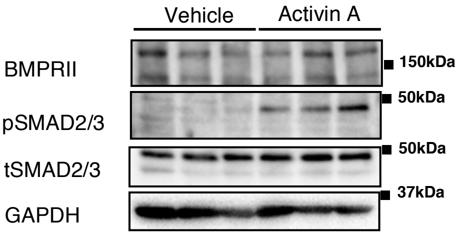
Vehicle
Activin A
BMPRII
150kDa
pSMAD2/3
50kDa
tSMAD2/3
50kDa
GAPDH
37kDa

e

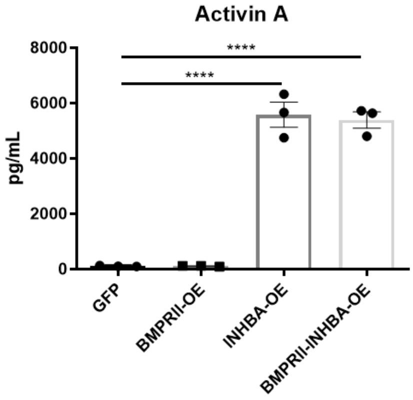
Activin A
pg/mL
8000
6000
4000
2000
0
****
****
GFP
BMPRII-OE
INHBA-OE
BMPRII-INHBA-OE

f

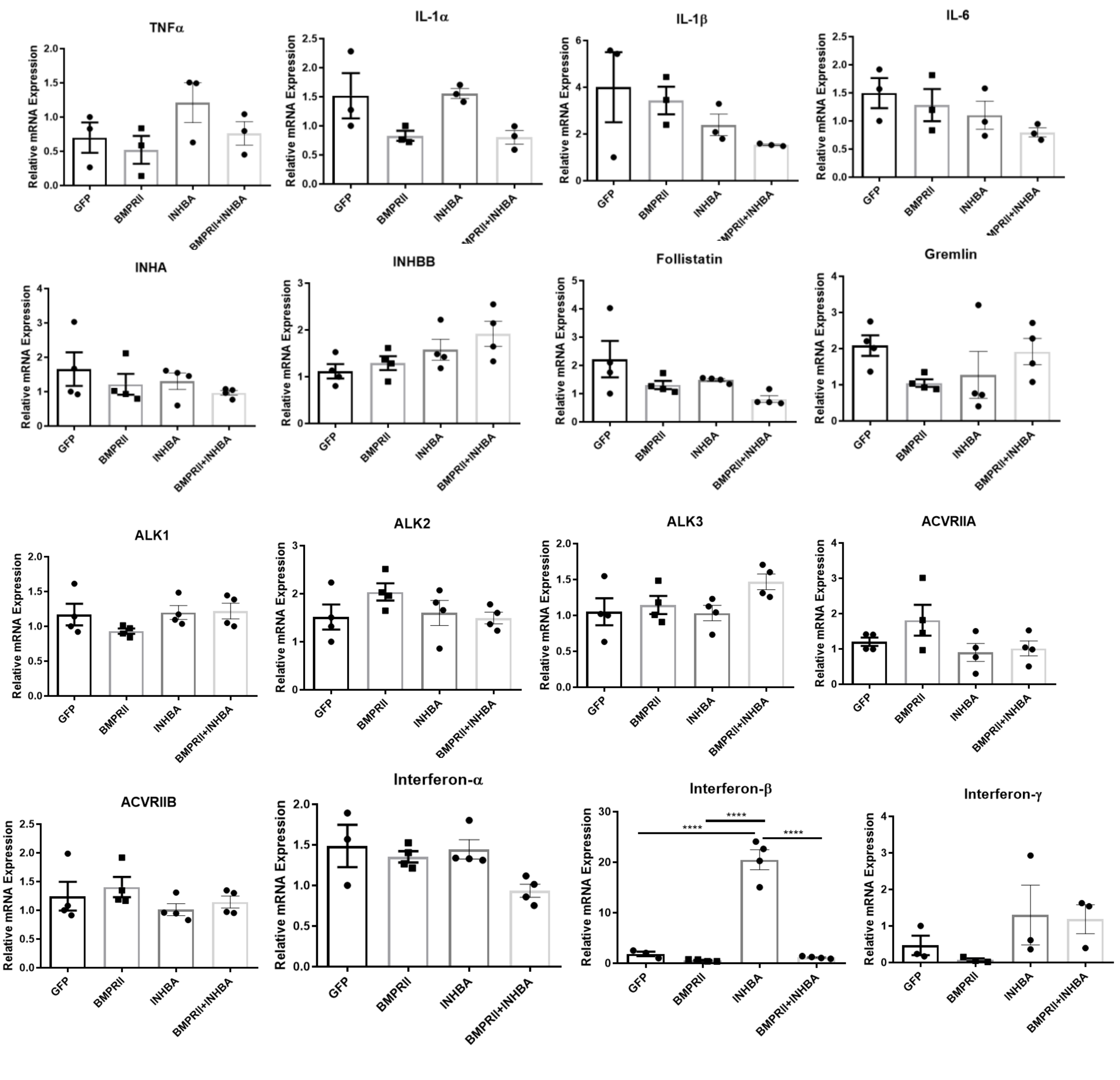

Supplementary Fig. 2. Biological effects of ActA in PAECs.

a, Immunoblotting for BMPRII, and representative images for Matrigel tube-formation assay and TUNEL staining for apoptosis were shown. Bars: 200 μm. PAECs without retrovirus transfection, transfected with empty virus envelop (MOCK), or with retrovirus carrying GFP were examined (n = 4 in each group for immunoblotting; n = 3 in each group for tube-formation and apoptosis assay). **b,** Immunoblottings for phosphorylated SMAD1/5 and β-actin in PAECs treated with either vehicle or recombinant BMP-4 (20 ng/mL) for 15 minutes were shown (n = 6 in each group). **c,** Immunoblottings for SMAD2/3 and GAPDH in PAECs treated with either vehicle or recombinant ActA (20 ng/mL) for 15 minutes (n = 6 in each group). **d,**

Immunoblottings for BMPRII, SMAD2/3, and GAPDH in PASMCs treated with either vehicle or recombinant ActA (20 ng/mL) for 15 minutes (n = 3 in each group). **e,** ActA concentration in the culture medium of PAECs transfected with GFP, BMPRII, INHBA, or BMPRII and INHBA was measured by ELISA (n = 3 in each group). **f,** Quantitative PCR analysis for *TNF-α*, *IL-1α*, *IL-1β*, *IL-6*, *INHA*, *INHBB*, *follistatin*, *gremlin-1*, *ALK1*, *ALK2*, *ALK3*, *ACVRIIA*, *ACVRIIB*, *interferon-α*, *interferon-β* and *interferon-γ* in PAECs transfected with either GFP, BMPRII, INHBA, or BMPRII and INHBA (n = 3 in each group for *TNF-α*, *IL-1α*, *IL-1β*, *IL-6,* and interferon-γ; n = 4 in each group for *INHA*, *INHBB*, *follistatin*, *gremlin-1*, *ALK1*, *ALK2*, *ALK3*, *ACVRIIA*, *ACVRIIB*, *interferon-α*, and *interferon-β*). ****$P < 0.0001$; n.s., not significant ($P > 0.05$). Data are presented as the mean ± SEM. Exact P values are shown in the Source Data file. One-way ANOVA with Tukey’s post-hoc test was used to compare between groups for (a, e, and f).

a

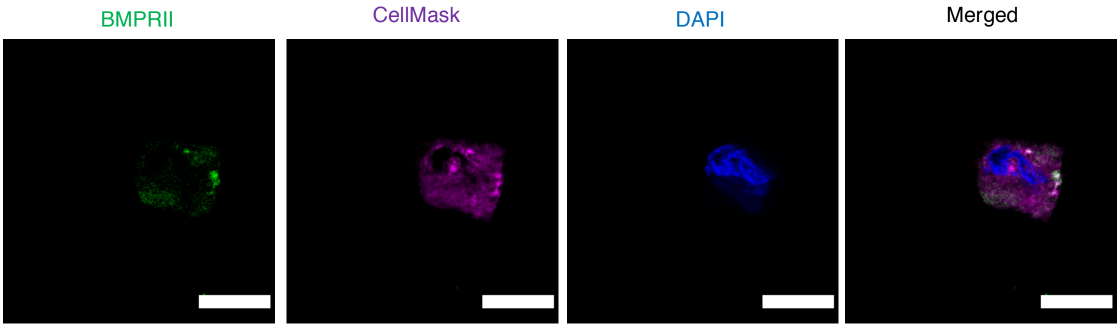

b

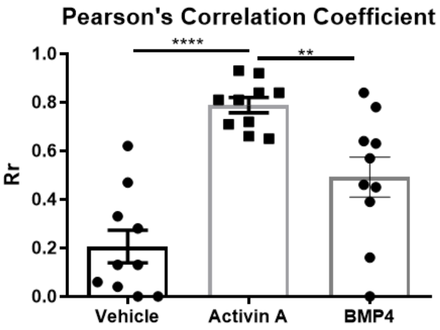

c

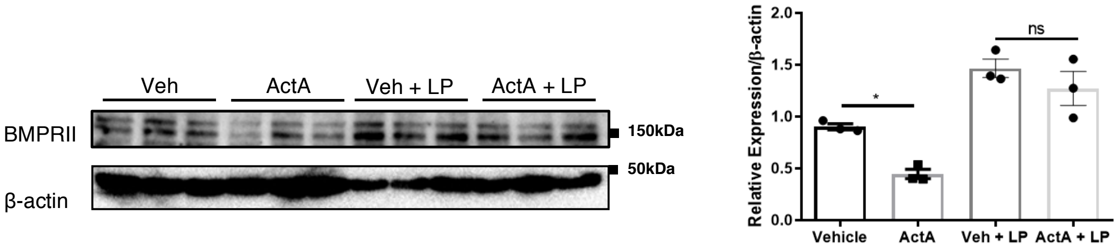

Supplementary Fig. 3. BMPRII trafficking mediated by ActA.

a, Representative images for BMPRII-GFP and plasma membrane-staining in PAECs. Plasma membrane was stained by using CellMask. Bars: 20 µm. **b,** Quantification of BMPRII-GFP and LysoTracker colocalisation in PAECs treated with either vehicle, ActA (20 ng/mL) or BMP-4 (20 ng/mL) assessed by Pearson's Correlation Coefficient (n = 10 cells for each group). **c,** Representative immunoblots and densitometry analysis for BMPRII in PAEC treated with either vehicle (Veh) or ActA for 30 min in the presence of cycloheximide (n = 3 in each group). Some cells were pretreated with leupeptin (LP) for 2 h before the stimulation. $^{*}P < 0.05$, $^{**}P < 0.01$, $^{***}P < 0.001$, $^{****}P < 0.0001$, n.s., not significant (P > 0.05). Data are presented as the mean ±

SEM. Exact P values are shown in the Source Data file. One-way ANOVA with Tukey's post-hoc test was used to compare between groups for all figures.

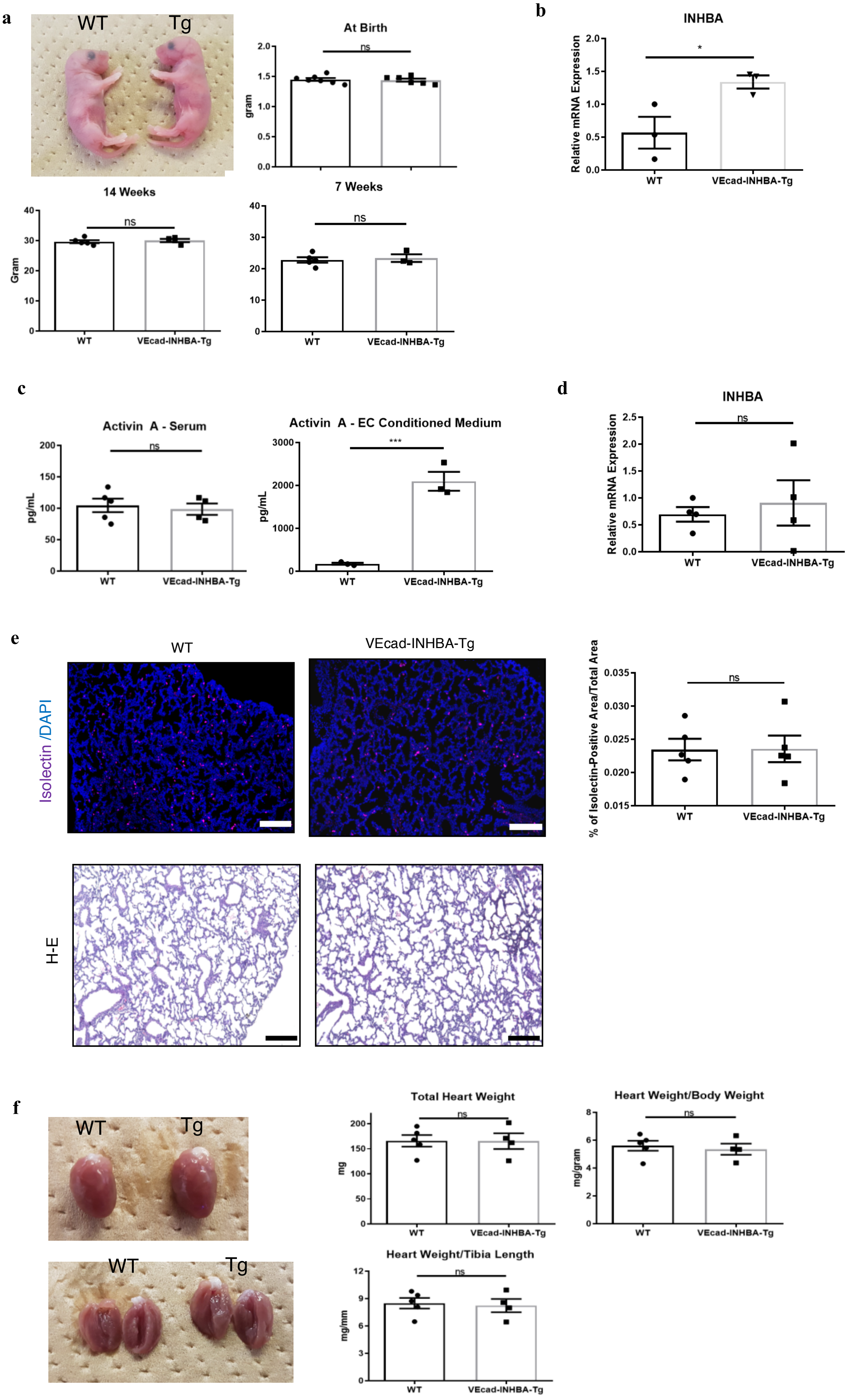
a
WT
Tg
At Birth
ns
gram
14 Weeks
Gram
7 Weeks
WT
VEcad-INHBA-Tg
b
INHBA
Relative mRNA Expression
*
c
Activin A - Serum
pg/mL
Activin A - EC Conditioned Medium
***
d
INHBA
e
VEcad-INHBA-Tg
Isolectin /DAPI
% of Isolectin-Positive Area/Total Area
H-E
f
Total Heart Weight
mg
Heart Weight/Body Weight
mg/gram
Heart Weight/Tibia Length
mg/mm

g

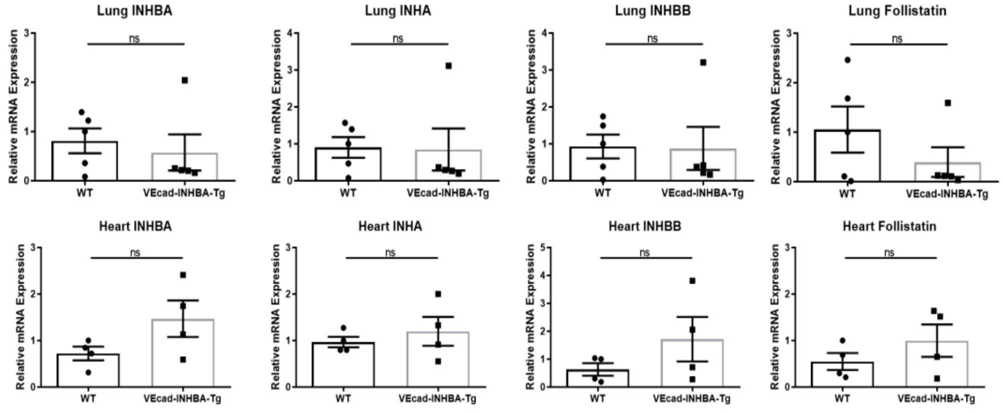

Supplementary Fig. 4. Phenotypic analyses for VEcad-INHBA-Tg mice.

a, Representative picture of VEcad-INHBA-Tg and WT littermate mice at birth and body weight of the mice at birth (n = 7 in each group), 7 weeks old (n = 4 in each group), and 14 weeks old (n = 5 in each group). **b,** Quantitative PCR analysis for *INHBA* in ECs isolated from the lungs of WT and VEcad-INHBA-Tg mice (n = 3 in each group). **c,** Serum levels of ActA in VEcad-INHBA-Tg and WT littermate mice were measured by ELISA (left; n = 5 for WT; n = 4 for Tg). ActA concentration in the conditioned medium derived from lung ECs isolated from VEcad-INHBA-Tg mice and WT littermate mice were also measured (right; n = 3 in each group). **d,** Quantitative PCR analysis for INHBA in bone marrow cells isolated from VEcad-INHBA-Tg mice and WT littermate mice (n = 4 in each group). **e,** Representative images for isolectin- and HE-staining in lung sections of VEcad-INHBA-Tg and WT littermate mice at birth. Isolectin-positive blood vessels were quantified (n = 5 in each group). Bars: 100 μm **f,** Representative macroscopic images of the heart of VEcad-INHBA-Tg and WT littermate mice. Heart weight in these mice was also analyzed (n = 5 for WT; n = 4 for Tg). **g,** Quantitative PCR analysis for *INHBA*, *INHA*, *INHBB*, and *follistatin* in the lungs and heart of VEcad-INHBA-Tg and WT littermate mice (n = 5 in each group for lung; n = 4 in each group for heart). *$P < 0.05$, **$P < 0.01$, ***$P < 0.001$, ****$P < 0.0001$, n.s., not significant ($P > 0.05$). Data are presented as the mean ± SEM. Exact P values are shown in the Source Data file. Two-sided Student's *t*-test was used to compare between the groups for all panels.

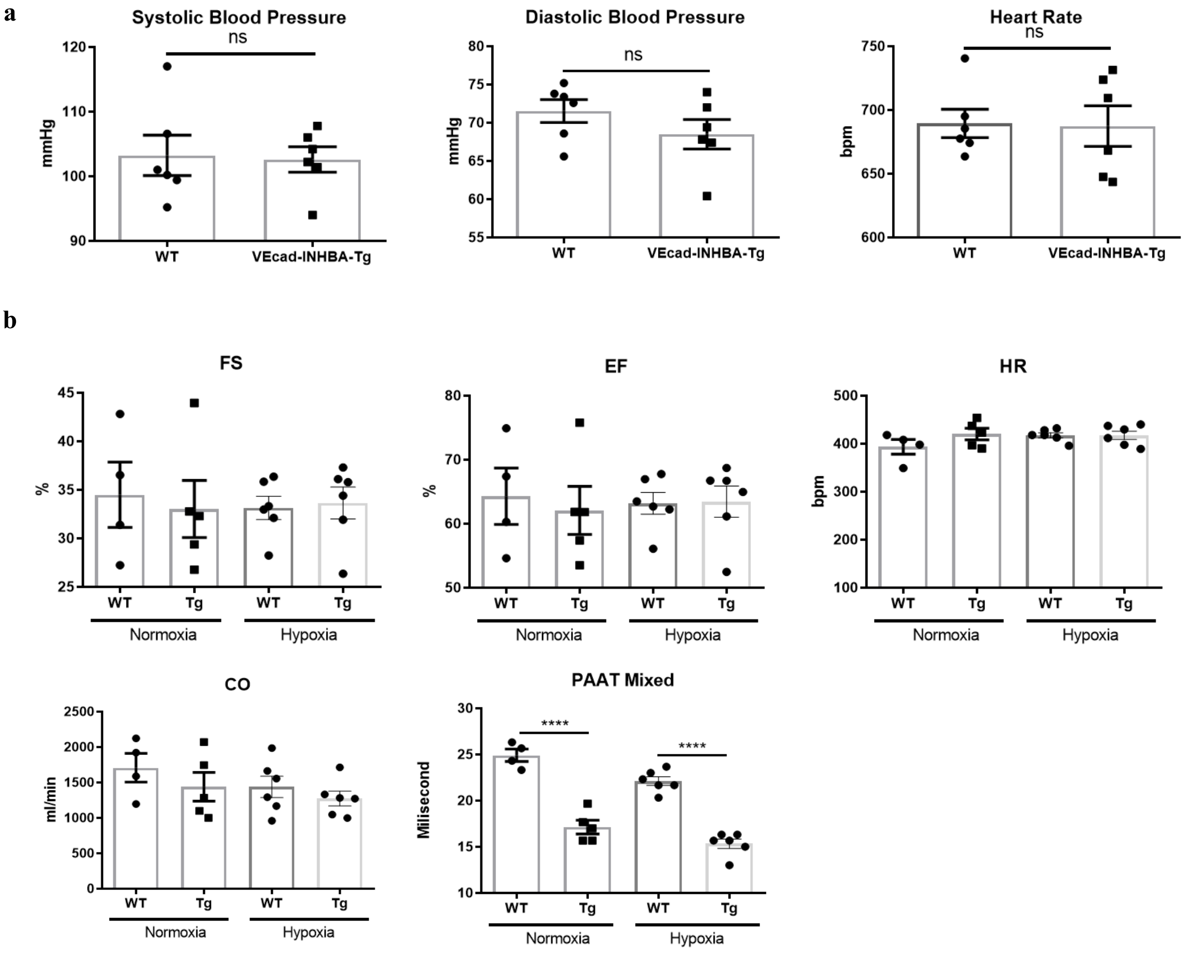

Supplementary Fig. 5. Hemodynamic characteristics in VEcad-INHBA-Tg mice. **a,** The systolic blood pressure, diastolic blood pressure, and heart rate in WT and VEcad-INHBA-Tg mice were measured (n = 6 in each group) under normoxic condition. **b,** Echocardiographic parameters, including fractional shortening (FS), ejection fraction (EF), heart rate (HR), cardiac output (CO), and pulmonary artery acceleration time (PAAT) in the WT and VEcad-INHBA-Tg mice under either normoxic or hypoxic condition (n = 4 for normoxia WT; n = 5 for normoxia Tg; n = 6 for hypoxia WT and hypoxia TG). ****$P < 0.0001$, n.s., not significant ($P > 0.05$). Data are presented as the mean ± SEM. Exact P values are shown in the Source Data file. Two-sided Student's *t*-test was used to compare between the groups (a). One-way ANOVA with Tukey's post-hoc test was used to compare between groups for figures (b).

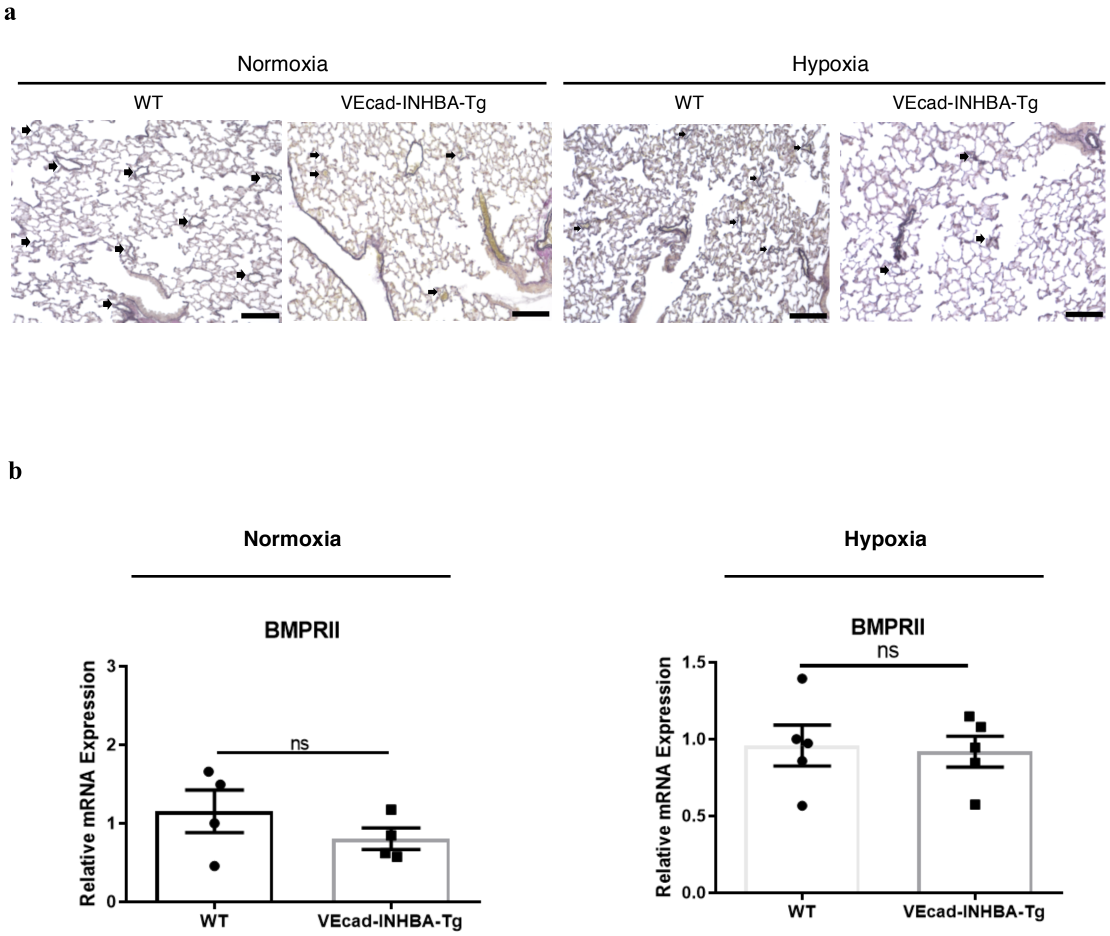

Supplementary Fig. 6. Analysis of the Lungs in VEcad-INHBA-Tg mice.

a, Representative images for Elastica Van Gieson-staining in lung sections prepared from VEcad-INHBA-Tg and WT littermate mice under either normoxic or hypoxic condition. Arrows indicate the distal arteries that were counted. Bars: 100 um. **b,** Quantitative RT-PCR analysis for *BMPRII* in the lungs of WT and VEcad-INHBA-Tg mice under either normoxic or hypoxic condition (n = 5 in each group). n.s., not significant (P > 0.05). Data are presented as the mean ± SEM. Exact P values are shown in the Source Data file. Two-sided Student's *t*-test was used to compare between the groups (b).

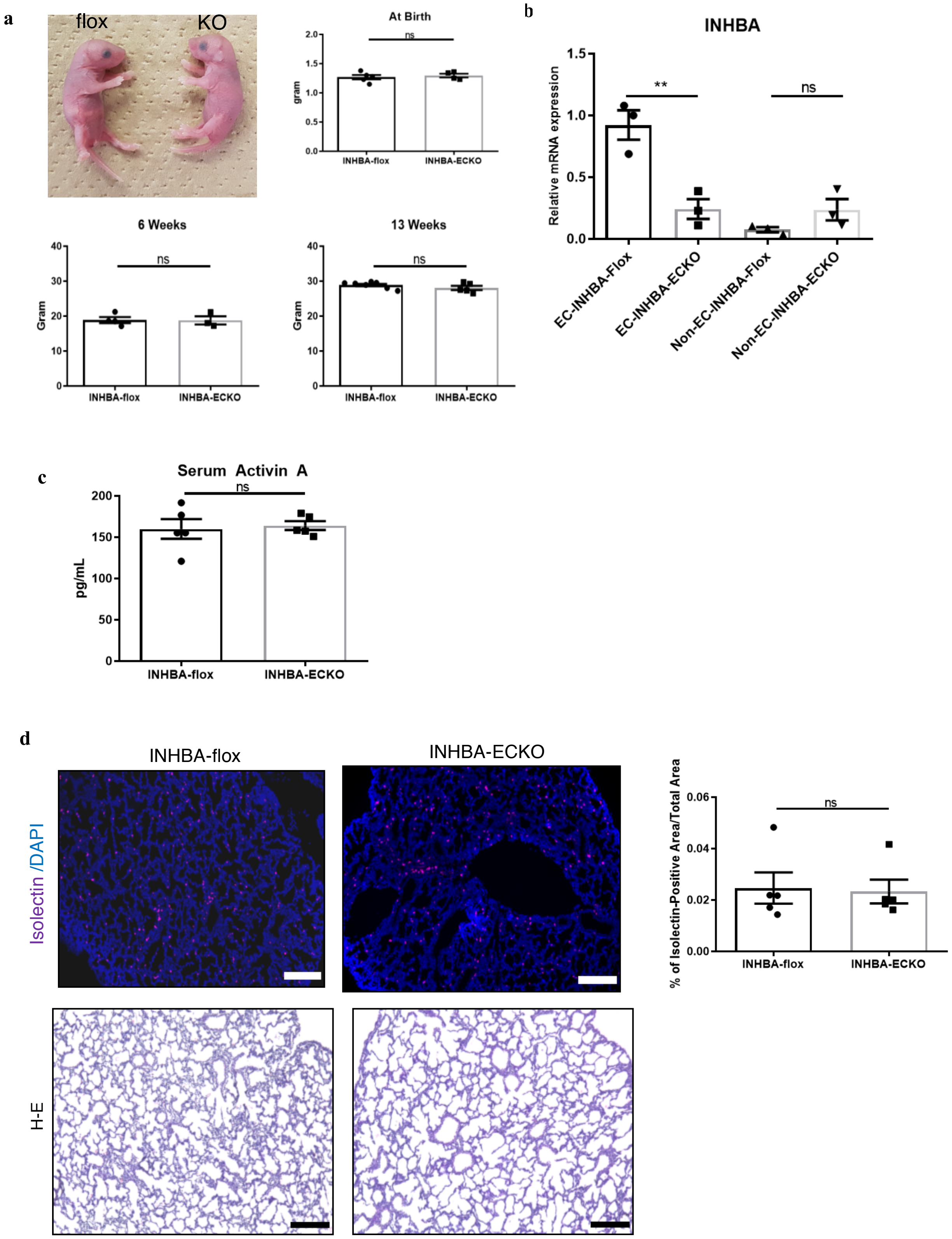

a
flox
KO
At Birth
gram
2.0
1.5
1.0
0.5
0.0
ns
INHBA-flox
INHBA-ECKO
6 Weeks
Gram
40
30
20
10
0
ns
INHBA-flox
INHBA-ECKO
13 Weeks
Gram
40
30
20
10
0
ns
INHBA-flox
INHBA-ECKO
b
INHBA
Relative mRNA expression
1.5
1.0
0.5
0.0
**
ns
EC-INHBA-Flox
EC-INHBA-ECKO
Non-EC-INHBA-Flox
Non-EC-INHBA-ECKO
c
Serum Activin A
pg/mL
200
150
100
50
0
ns
INHBA-flox
INHBA-ECKO
d
INHBA-flox
INHBA-ECKO
Isolectin /DAPI
H-E
% of Isolectin-Positive Area/Total Area
0.06
0.04
0.02
0.00
ns
INHBA-flox
INHBA-ECKO

e

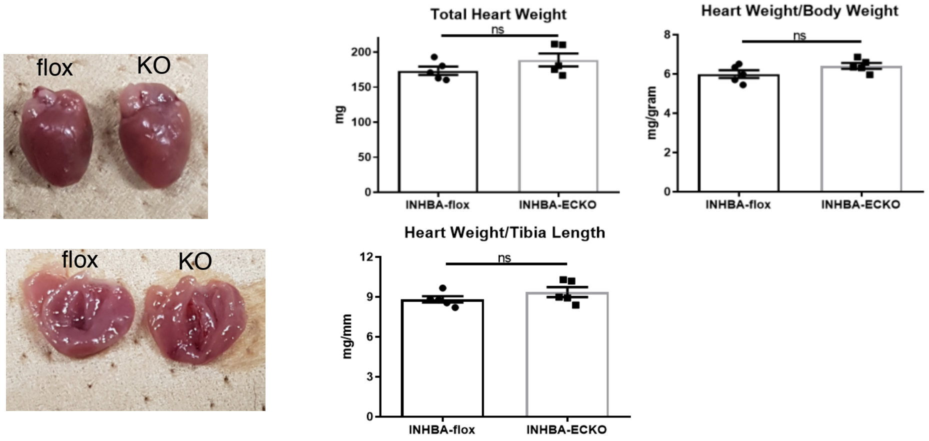

f

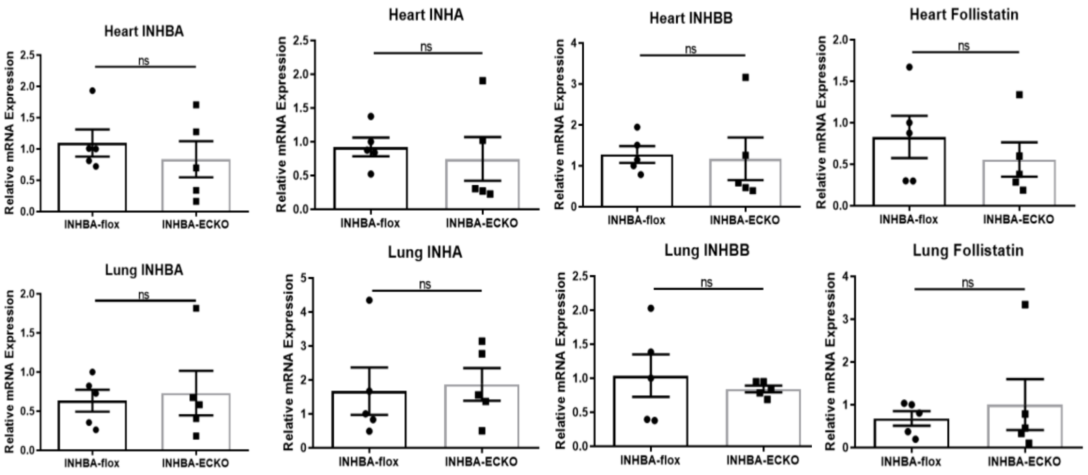

Supplementary Fig. 7. Phenotypic analyses for INHBA-ECKO mice.

a, Representative picture of INHBA-ECKO and INHBA-flox littermate mice at birth, and body weight of the mice at birth (n = 5 in each group), 6 weeks old (n = 4 in each group), and 13 weeks old (n = 7 for flox; n = 5 for ECKO). **b,** Quantitative PCR analysis for INHBA in ECs and non-ECs isolated from the lungs of INHBA-ECKO and INHBA-flox mice (n = 3 in each group). **c,** Serum levels of ActA were measured by ELISA in INHBA-ECKO and INHBA-flox mice (n = 5 in each group). **d,** Representative images for isolectin- and HE-staining of lung sections prepared from INHBA-ECKO and INHBA-flox littermate mice at birth. Isolectin-positive blood vessels were quantified (n = 5 in each group). Bars: 100 μm. **e,** Representative images of the heart of INHBA-ECKO and INHBA-flox littermate mice. Heart weight was also analyzed (n = 5 in each group). **f,** Quantitative PCR analysis for *INHBA*, *INHA*, *INHBB*,

and *follistatin* in the lungs and heart of INHBA-ECKO and INHBA-flox littermate mice (n = 5 each group). $^{**}P < 0.01$. n.s., not significant ($P > 0.05$). Data are presented as the mean ± SEM. Exact P values are shown in the Source Data file. Two-sided Student's *t*-test was used to compare between the groups for all panels.

a

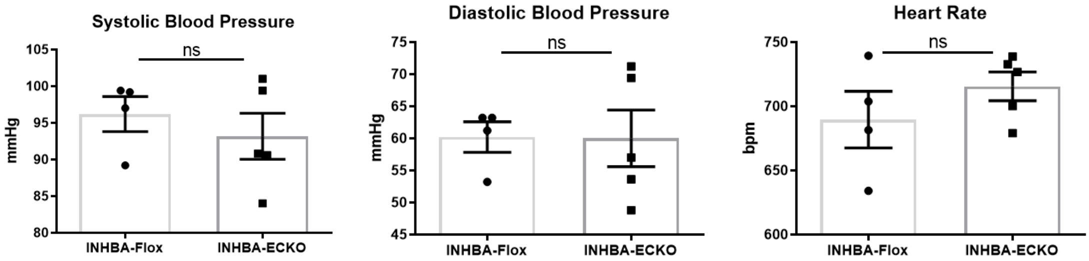

b

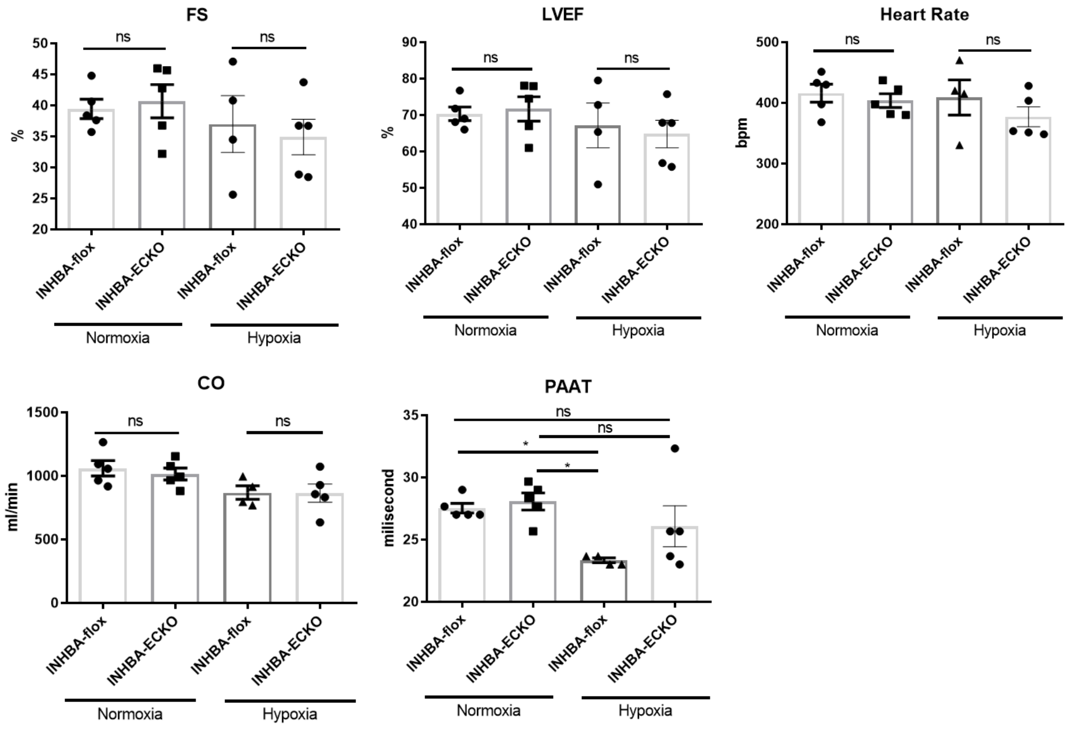

Supplementary Fig. 8. Hemodynamics in INHBA-ECKO mice.

a, The systolic blood pressure, diastolic blood pressure, and heart rate were measured in INHBA-flox and INHBA-ECKO mice under normoxic condition (n = 4 for flox; n = 5 for ECKO). **b,** Echocardiographic parameters in INHBA-flox and INHBA-ECKO mice under either normoxic or hypoxic condition (n = 5 for normoxic flox, normoxic ECKO, hypoxic ECKO; n = 4 for hypoxic flox). **P* < 0.05. n.s., not significant (P > 0.05). Data are presented as the mean ± SEM. Exact P values are shown in the Source Data file. Two-sided Student's *t*-test was used to compare between the groups for all panels.

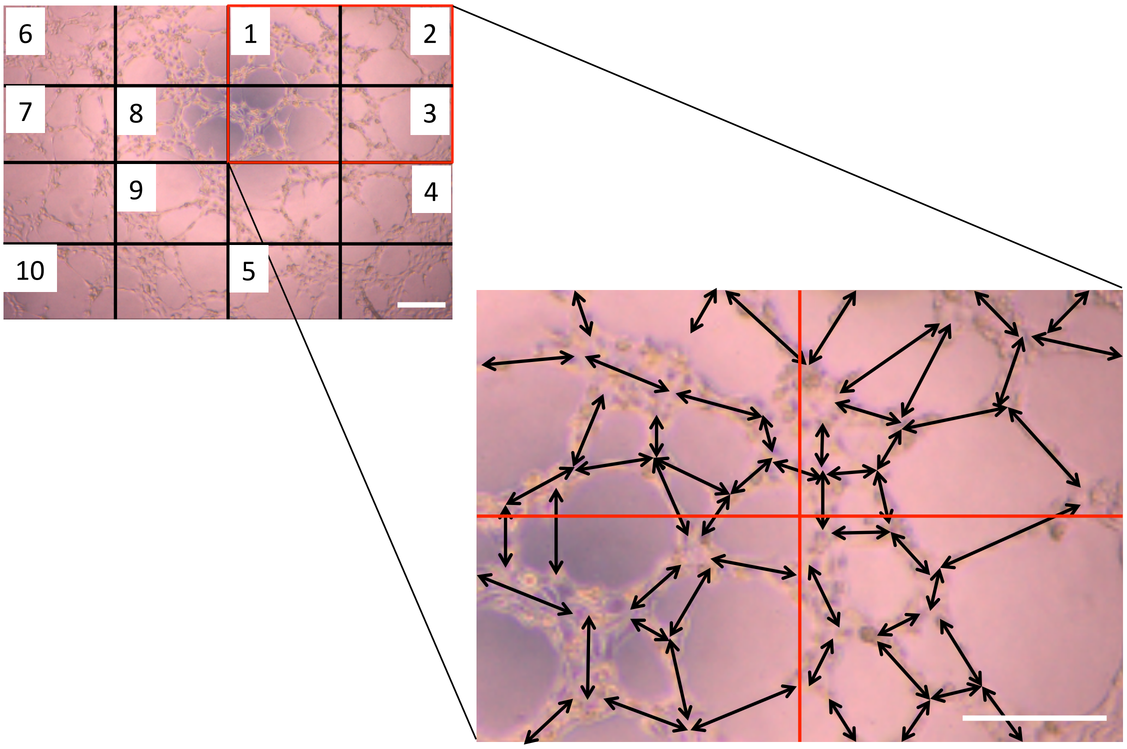

Supplementary Fig. 9. Measurements for tube lengths.

An image (x4) obtained from each well was divided into 16 rectangles, and the lengths of tubes between the branching junctions (indicated by lines with arrow heads at both ends) were measured and summed in a single rectangle in the image. Measurements were performed in 10 randomly chosen rectangles, and then values from each rectangle were averaged. Bars: 200 µm

Supplementary Table 1. List of Antibodies

Antibody Name	Manufacturer	Catalogue Number	Dilution
Anti-BMPRII	BD Biosiences	612292	1:250
Anti-phospho SMAD 1/5	Cell Signaling Technology	9516	1:1000
Anti-βactin	Cell Signaling Technology	4970	1:1000
Anti-GAPDH	Cell Signaling Technology	2118	1:1000
Anti-phospho Serine/Threonine	Abcam	ab117253	1:1000
Anti-Rabbit IgG, HRP-linked	Cell Signaling Technology	7074	1:3000
Anti-mouse IgG, HRP-linked	Cell Signaling Technology	7076	1:3000
Anti-von Willebrand Factor	Abcam	ab6994	1:200
Anti-Actin, α-Smooth Muscle, FITC-linked	Sigma-Aldrich	F3777	1:200
Anti-Rabbit Fluorescence-labeled IgG Cy3	Abcam	ab6939	1:200
Anti-phospho SMAD 2/3	Cell Signaling Technology	8828	1:1000
Anti-SMAD 2/3	Cell Signaling Technology	3102	1:1000

Supplementary Table 2. List of qPCR Primers

Gene	
Human	
BMPRII	Forward: 5'-caaatctgtgagcccaacagtcaa-3'
	Reverse: 5'-gaggaagaataatctggataaggaccaat-3'
Id1	Forward: 5'-ctgctctacgacatgaacggc-3'
	Reverse: 5'-tgacgtgctggagaatctcca-3'
INHBA	Forward: 5'-ggacgttcggattgcctgtgagcagtg-3'
	Reverse: 5'- gaggaaaggtctgtgcgactgctcct-3'
INHA	Forward: 5'-agccacagatgccagctgtgaggac-3'
	Reverse: 5'- ggaaccacagctgggctgaagtcac-3'
INHBB	Forward: 5'-cctgcctgaacgcacatgacatagca-3'
	Reverse: 5'-ccagtttcgttctagtgtgggtcaac-3'
BMPRII-SalI	Forward:5'- tcagtccacgggagagaagacagc-3'
	Reverse: 5'-actcgacagacagttcattcctatatctt-3'
Human-mouse INHBA common sequence	Forward: 5'-gctggaatgactggatcattgctcc-3'
	Reverse: 5'-acargggtctcagcttggtgggcaca-3'
TNFα	Forward: 5'-tgcactttggagtgatcggc-3'
	Reverse: 5'-ctcagcttgagggtttgctac-3'
IL-1α	Forward: 5'-ggtcaccaaattctacttccaggaggac-3'
	Reverse: 5'-gtgaccaggttgttgtgacgccttc-3'
IL-1β	Forward: 5'-agctgtacccagagagtcctgtgctga-3'
	Reverse: 5'-aggagagagctgactgtcctggctgatg-3'
IL-6	Forward: 5'-gaagctgcaggcacagaaccagtggc-3'
	Reverse: 5'-ctgaccagaagaaggaatgcccatt-3'
Gremlin-1	Forward: 5'-tcactctcggtcccgctga-3'
	Reverse: 5'- ctgtgcggctcatactgtca-3'
Follistatin	Forward: 5'-cctgagaaaggctacctgcc-3'
	Reverse: 5'- tcttcacaggactttgctttgat-3'
Interferon-α	Forward: 5'-cttgtgcctgggaggttgtc-3'
	Reverse: 5'-tagcaggggtgagagtctttg-3'
Interferon-β	Forward: 5'-agtaggcgacactgttcgtg-3'
	Reverse: 5'-gcctcccattcaattgccac-3'
Interferon-γ	Forward: 5'- gctgttactgccaggaccc-3'
	Reverse: 5'-ttttctgtcactctcctctttcc-3'

ALK1	Forward: 5’-gaggcaggagaagcagcgt-3’
	Reverse: 5’-gccttttcccacacactcca-3’
ALK2	Forward: 5’-tggagatttgaacgctgcttg-3’
	Reverse: 5’-cttaccaatgctccaggcttgc-3’
ALK3	Forward: 5’-ctggggtccggacttatgaaa-3’
	Reverse: 5’-tacgactcctccaagatgtgg-3’
ACVRIIA	Forward: 5’-gtgtctgggttgaaggaggtt-3’
	Reverse: 5’-aactttgcagcagctcccat-3’
ACVRIIB	Forward: 5’-actacaacgccaactgggag-3’
	Reverse: 5’- ccacacactcctgcctatcg-3’
Mouse	
BMPRII	Forward: 5’-gagccctcccttgacctg-3’
	Reverse: 5’-gtatcgaccccgtccaatc-3’
ID1	Forward: 5’-cccactggaccgatccgcca-3’
	Reverse: 5’-tgctcttcggttccccagggg-3’
INHBA-coding region	Forward: 5’-gaaggcaaccacacgacttttgctgc-3’
	Reverse: 5’-ctctggctgagagttaggtccatccttc-3’
INHA	Forward: 5’-gatcctggaataaggcgtctgcctc-3’
	Reverse: 5’-ctcggcagctggctggtcctcaca-3’
INHBB	Forward: 5’-ggctgttctgaggagctgctcagctg-3’
	Reverse: 5’-ctggacacacgtagacctgcaagtgc-3’
Follistatin	Forward: 5’-tcgctgctctctctgcgat-3’
	Reverse: 5’-ccgtttcttccgagatggagttg-3’

Supplementary Table 3. List of Reagents and Recombinant Proteins

**Name**	**Manufacturer**	**Catalogue Number**
Human activin A	R&D Systems	338-AC
Human follistatin	R&D Systems	4889-FN
Cycloheximide	Sigma-Aldrich	01810
Bafilomycin A1	Sigma-Aldrich	B1793
Pitstop 2	Sigma-Aldrich	SML1169
Polybrene/Hexadimethrine Bromide	Sigma-Aldrich	H9268
Leupeptin	Fujifilm Wako Chemicals	336-40413
