# Supplementary material for: An endothelial activin A-bone morphogenetic protein receptor type 2 link is overdriven in pulmonary hypertension
Source: Nat Commun. 2021 Mar 19;12:1720. doi: 10.1038/s41467-021-21961-3 (PMC7979873; doi:10.1038/s41467-021-21961-3)
Supplement: Supplementary file 3 — Reporting Summary [file 41467_2021_21961_MOESM3_ESM.pdf]

nature research

Corresponding author(s): Koji Ikeda

Reporting Summary

Nature Research wishes to improve the reproducibility of the work that we publish. This form provides structure for consistency and transparency in reporting. For further information on Nature Research policies, see Authors & Referees and the Editorial Policy Checklist.

Statistical parameters

When statistical analyses are reported, confirm that the following items are present in the relevant location (e.g. figure legend, table legend, main text, or Methods section).

n/a	Confirmed	
☐	☒	The exact sample size (n) for each experimental group/condition, given as a discrete number and unit of measurement
☐	☒	An indication of whether measurements were taken from distinct samples or whether the same sample was measured repeatedly
☐	☒	The statistical test(s) used AND whether they are one- or two-sided<br>*Only common tests should be described solely by name; describe more complex techniques in the Methods section.*
☒	☐	A description of all covariates tested
☐	☒	A description of any assumptions or corrections, such as tests of normality and adjustment for multiple comparisons
☐	☒	A full description of the statistics including central tendency (e.g. means) or other basic estimates (e.g. regression coefficient) AND variation (e.g. standard deviation) or associated estimates of uncertainty (e.g. confidence intervals)
☐	☒	For null hypothesis testing, the test statistic (e.g. F, t, r) with confidence intervals, effect sizes, degrees of freedom and P value noted<br>*Give P values as exact values whenever suitable.*
☒	☐	For Bayesian analysis, information on the choice of priors and Markov chain Monte Carlo settings
☒	☐	For hierarchical and complex designs, identification of the appropriate level for tests and full reporting of outcomes
☒	☐	Estimates of effect sizes (e.g. Cohen's d, Pearson's r), indicating how they were calculated
☐	☒	Clearly defined error bars<br>*State explicitly what error bars represent (e.g. SD, SE, CI)*

Our web collection on statistics for biologists may be useful.

Software and code

Policy information about availability of computer code

Data collection	No software was used.
Data analysis	Statistical analysis was performed using the GraphPad 8.0 software.

For manuscripts utilizing custom algorithms or software that are central to the research but not yet described in published literature, software must be made available to editors/reviewers upon request. We strongly encourage code deposition in a community repository (e.g. GitHub). See the Nature Research guidelines for submitting code & software for further information.

Data

Policy information about availability of data

All manuscripts must include a data availability statement. This statement should provide the following information, where applicable:
- Accession codes, unique identifiers, or web links for publicly available datasets
- A list of figures that have associated raw data
- A description of any restrictions on data availability

The authors declare that all data supporting the findings of this study are available within the paper and its supplementary information files. The source data underlying Figs. 1b-c, 2a-c, 3a-g, 3i, 4a, 4c-d, 4g, 5a-b, 5d-h, 6a-b, and 6d-i are provided as the Source Data file. The DNA microarray data have been deposited to the Gene Expression Omnibus with the dataset identifier GSE156225 (https://www.ncbi.nlm.nih.gov/geo/query/acc.cgi?acc=GSM4727144) and GSE156233 (https://

www.ncbi.nlm.nih.gov/geo/query/acc.cgi?acc=GSE156233). All remaining data will be available from the corresponding author upon reasonable request.

Field-specific reporting

Please select the best fit for your research. If you are not sure, read the appropriate sections before making your selection.

- [x] Life sciences
- [ ] Behavioural & social sciences
- [ ] Ecological, evolutionary & environmental sciences

For a reference copy of the document with all sections, see nature.com/authors/policies/ReportingSummary-flat.pdf

Life sciences study design

All studies must disclose on these points even when the disclosure is negative.

Sample size	We did not statistically determine the sample size, but common sample size for each group for this type of experimental work in literature was chosen. Basically, more than three cells/samples were used for in vitro experiments, while more than five animals/samples were used for in vivo experiments.
Data exclusions	We did not exclude any data from the analysis.
Replication	For all the data, experiments were replicated for 2-3 independent times.
Randomization	We used randomization whenever possible. Specifically, we randomly determined the allocation of animals (normoxia or hypoxia) and allocation of cells (GFP-transfection or INHBA-transfection; vehicle group or Activin A group; vehicle group or Follistatin group; vehicle group or BMP-4 group or Activin A group; vehicle group or Bafilomycin group; vehicle group or PitStop group; etc.). Also, we captured images of lung histologies at randomly chosen fields.
Blinding	Because single researcher (GRTR) has performed the majority of experiments, investigators were not blinded to sample allocation during experiments and outcome assessment. However, most of data are objective and there is no room for biased evaluation. Also, efforts to avoid biased evaluation were made by using multiple biologically independent samples as well as by choosing multiple ROI for data acquisition.

Reporting for specific materials, systems and methods

Materials & experimental systems

n/a	Involved in the study	
x		Unique biological materials
	x	Antibodies
	x	Eukaryotic cell lines
x		Palaeontology
	x	Animals and other organisms
	x	Human research participants

Methods

n/a	Involved in the study	
x		ChIP-seq
x		Flow cytometry
x		MRI-based neuroimaging

Antibodies

Antibodies used	BMPRII: BD Biosiences #612292, pSMAD 1/5: CST #9516, pSerine-Threonine: abcam #ab117253, Beta-Actin: CST #4970,GAPDH: CST #2118, von Willebrand Factor: abcam #ab6994, alpha-SMA: sigma #F3777, pSMAD2/3: CST #8828, SMAD2/3: CST #3102, HRP-rabbit IgG: CST #7074, HRP-mouse IgG: CST #7076, Cy3-rabbit IgG: abcm #ab6939
Validation	According to the data sheet, all the antibodies used for immunoblotting can detect both mouse and human target proteins. In addition, von Willebrand Factor and alpha-SMA antibody are both suitable for mouse immunofluorescent staining in accordance to the infromation from data sheet.

Eukaryotic cell lines

Policy information about cell lines

Cell line source(s)	GP2-293
Authentication	Cells were obtained from Clontech.

Mycoplasma contamination	We regularly perform the mycoplasma test for cultured cells, and confirm no mycoplasma contamination.
Commonly misidentified lines (See ICLAC register)	No misidentified lines were used in this study.

Animals and other organisms

Policy information about studies involving animals; ARRIVE guidelines recommended for reporting animal research

Laboratory animals	Mice were housed in designated cages of sufficient size (1-3 mice in one cage) in animal facility in which the temperature (~23 °C) and humidity (~60 %) are regulated appropriately. Mice were maintained under a 12-h light/12-h dark cycle, and fed normal chow (containing 23.1% protein and 5.1% fat) with ad libitum access to water and food. Mice were regularly used for experiments at 8–12 weeks of age.
Wild animals	No wild animals were involved in this study.
Field-collected samples	No field-collected samples were involved in this study.

Human research participants

Policy information about studies involving human research participants

Population characteristics	Idiopathic PAH patients' characteristics: n=12, age mean= 38.5±11.5, 3 males and 9 females<br>Control specimens' characteristics: n=16, age mean=65,8±11.5, 10 males and 6 females
Recruitment	The study protocols were approved by the local ethics committee (CPP Ile-de-France VII, Le Kremlin-Bicêtre, France). All patients gave informed consent before the study. Human pulmonary ECs were isolated from lung explants of idiopathic PAH patients without BMPR2 mutation (n=12) or from lung specimens obtained during lobectomy or pneumonectomy at a distance from the tumor foci in control subjects (Hôpital Marie Lannelongue, Le Plessis Robinson, France) (n=16). Preoperative echocardiography was performed in these control patients to rule out PH and the absence of tumoral infiltration was retrospectively established in all tissue sections by the histopathological analysis.
